# CLOSE-Guided Pulmonary Vein Isolation to Treat Persistent Atrial Fibrillation: 1-Year Outcome

**DOI:** 10.3390/jcm12144698

**Published:** 2023-07-15

**Authors:** Philippe Taghji, Jean-Claude Deharo, Sana Amraoui, Sok-Sithikun Bun

**Affiliations:** 1 Electrophysiology Unit, Cardiology Department, La Timone University Hospital, 13005 Marseille, France; 2 Electrophysiology Unit, Cardiology Department, American Hospital of Paris, 92200 Neuilly-sur-Seine, France; 3 Electrophysiology Unit, Cardiology Department, Pasteur University Hospital, 06000 Nice, France

**Keywords:** CLOSE-guided ablation, persistent atrial fibrillation, pulmonary vein isolation, atrial fibrillation, single-procedure arrhythmia-free survival

## Abstract

Background: CLOSE-guided pulmonary vein isolation (PVI) is based on contiguous and optimized (Ablation Index-guided) radiofrequency lesions. The efficacy of CLOSE-guided PVI in persistent atrial fibrillation (AF) treatment has been poorly evaluated. Methods: In two centers, 50 patients eligible for persistent AF ablation underwent CLOSE-guided PVI (Ablation Index ≥ 450 at the anterior wall, ≥300 at posterior wall, intertag distance ≤ 6 mm). If PVI failed to restore sinus rhythm (SR), electrical cardioversion (ECV) was performed. Atrial substrate modification (ASM) was performed only if PVI and ECV failed to restore SR. Recurrence was defined as any recorded episode of AF, atrial tachycardia (AT) or atrial flutter (AFL) > 30 s on Holter electrocardiographs at 3, 6 and 12 months. Results: From the 50 patients (64 ± 10 years, 14% long-standing persistent AF), SR was restored by ECV in 34 patients (68%) 56 ± 38 days prior to ablation. On the day of ablation, 42 patients (84%) were on class I-III anti-arrhythmic drug therapy (ADT) and the rhythm was AF in 23/50 patients. PVI was achieved in all patients; after PVI, ECV was required in 21 patients and ASM in 1 patient. The mean procedure time, radiofrequency time and fluoroscopy time were 141 ± 33 min, 23 ± 7 min and 7 ± 6 min, respectively. At 12 months, single-procedure freedom from AF/AT/AFL was 80%, with 19 patients (38%) receiving class I-III ADT. Conclusions: In a population of patients with persistent AF monitored with intermittent cardiac rhythm recordings, CLOSE-guided PVI resulted in high single-procedure arrhythmia-free survival at 1 year. Future large-scale studies involving continuous cardiac monitoring are necessary.

## 1. Introduction

Pulmonary vein isolation (PVI)—the cornerstone of catheter-based atrial fibrillation (AF) ablation [1]—is less successful for persistent AF than for paroxysmal AF, often requiring repeated procedures [2,3,4]. In Europe, approximately one third of AF ablation procedures are performed in patients with persistent AF [5]. However, ablation strategies based on anatomical and/or electrophysiological approaches are associated with poor outcomes in this specific population [6], highlighting the need for a safe, efficient and reproducible ablation technique that can be performed within a reasonable amount of time.

Contiguous lesion optimized pre-specified encircling (CLOSE)-guided PVI is an ablation technique aiming at creating contiguous and optimized (Ablation Index-guided) point-by-point radiofrequency (RF) lesions. The efficacy of CLOSE-guided PVI in treating the paroxysmal form of AF has been demonstrated in multicenter studies [7,8]. However, the efficacy of CLOSE-guided PVI to treat persistent forms of AF has been poorly evaluated.

In the present study, we aimed to: (1) evaluate 1-year clinical outcomes in a cohort of patients with persistent AF after CLOSE-guided PVI; and (2) describe the different forms of atrial tachyarrhythmia (ATA) recurrence after CLOSE-guided PVI.

## 2. Materials and Methods

### 2.1. Study Participants

**Figure 1:**
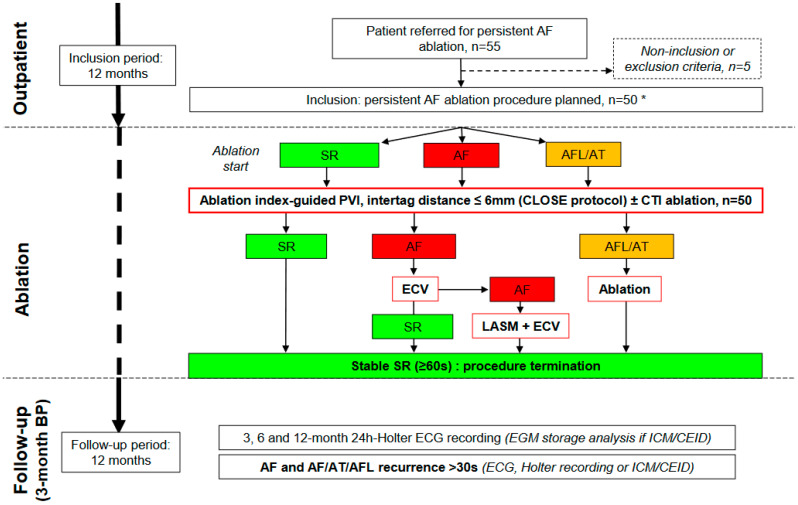
Study workflow. AF = atrial fibrillation; AFL= atrial flutter; AT = atrial tachycardia; BP = blanking period; CEID = cardiac electronic implantable device; CTI = cavotricuspid isthmus; ECG = electrocardiogram; ECV = electrical cardioversion; EGM = electrogram; ICM = insertable cardiac monitor; LASM = left atrium substrate modification; PVI = pulmonary veins isolation; SR = sinus rhythm. * Outpatient ECV was encouraged prior to ablation.

The workflow of this study is illustrated in Figure 1. From October 2020 to October 2021, consecutive patients aged 18–80 years who were referred for persistent AF catheter ablation at the two participating institutions (La Timone University Hospital, Marseille, FR, France; Pasteur University Hospital, Nice, FR, France) were evaluated and enrolled by 2 electrophysiologists (PT and SB). To be included in this study, patients were required to (1) have an ongoing uninterrupted episode of symptomatic persistent AF (continuous episode sustained beyond 7 days) documented by 12-lead electrocardiograms at the outpatient clinic visit and (2) be refractory, intolerant or unwilling to take class I or class III anti-arrhythmic drug therapy (ADT). Non-inclusion criteria were as follows: (1) long-standing persistent AF with ongoing uninterrupted AF episodes ≥ 3 years; (2) New York Heart Association functional class IV; (3) left ventricle ejection fraction ≤ 15%; (4) previous left atrium (LA) ablation; (5) previous mitral valve surgery; (6) congenital heart disease; (7) myocardial infarction or cardiac percutaneous intervention in the previous 6 months; (8) stroke or systemic embolism in the previous 6 months. All included patients underwent CLOSE-guided PVI followed by cavotricuspid isthmus (CTI) ablation if typical atrial flutter (AFL) had been documented prior to inclusion. All the patients gave their informed consent for the procedure. The study protocol (PADS 23-69) conforms to the ethical guidelines of the 1975 Declaration of Helsinki and was approved by the institutional human research committee.

### 2.2. Study Design

This was a prospective, single-arm, two-center observational study aimed at investigating the efficacy of CLOSE-guided PVI in subjects eligible for persistent AF ablation. Efficacy was defined as freedom from any recurrence of ATA episodes > 30 s over a 12-month follow-up period after ablation.

### 2.3. Scheduled Electrical Cardioversion Prior to Ablation

Between inclusion and ablation, scheduled electrical cardioversion (ECV) was encouraged in patients with a European Heart Rhythm Association functional class ≥ II. If scheduled ECV was performed, the results and the time between ECV and the day of ablation were collected.

### 2.4. Ablation Procedure

**Figure 2:**
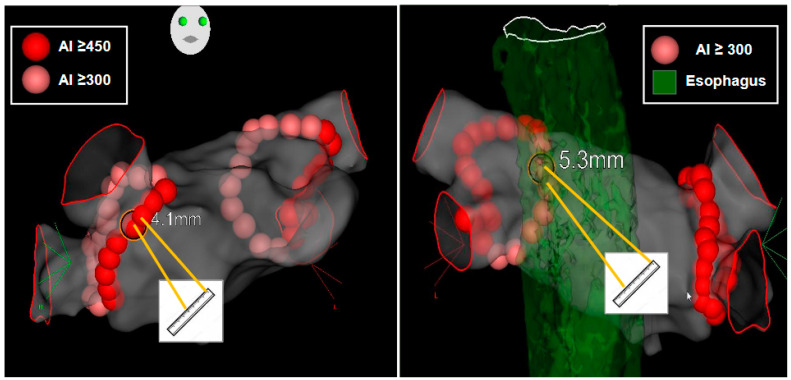
Region specific Ablation Index target with intertag distance. Left panel, RAO 30° view: AI target ≥ 450 au on the AW (red-colored tag), AI target ≥ 300 au on the PW and roof (pink-colored tag). Intertag distance target was ≤6 mm. Right panel, PA view: at the vicinity of esophagus (localized by preprocedure tomodensitometry or if esophageal probe temperature rise ≥ 38.5 °C), intertag distance target was ≤7 mm. RAO = right anterior oblique; AI = Ablation Index; au = arbitrary unit; AW = anterior wall; PW = posterior wall; PA = postero-anterior.

Data on rhythm at the beginning of the procedure (SR, AF, AT or AFL) were collected. The procedure was performed under general anesthesia or conscious sedation by two operators (PT, SB) at the two participating institutions. In the case of general anesthesia, esophageal temperature was monitored (SensiTherm^®^, St. Jude Medical, St. Paul, MN, USA). The femoral sheaths were introduced using ultrasound-guided venous puncture [9]. Intravenous heparin (100 IU/kg) was administered immediately after introducing the femoral sheaths and continuously infused (Activated Clotting Time *>* 350 s); in cases of uninterrupted direct oral anticoagulant intake, intravenous heparin was administered immediately after transeptal puncture. All patients underwent CLOSE-guided PVI as previously described (Figure 2) [10]. Real-time automated display of RF applications (Visitag, Biosense Webster, Inc) was used with predefined settings of catheter stability (3 mm for 3 s) and minimum contact force (CF; 30% of time > 3 g). RF was delivered in power-controlled mode (without ramping, tip temperature < 43 °C) using 35 W (posterior wall) and 45 W (anterior wall and roof) with an irrigation rate of 17 to 30 mL/min. RF was delivered until the Ablation Index (AI) reached ≥450 at the anterior wall and roof and ≥300 at the posterior wall (see Figure 1). In cases of intraesophageal temperature rising above 38.5 °C during posterior wall ablation or in the vicinity of the esophagus (localized on pre-procedural cardiothoracic tomodensitometry), RF delivery was stopped at an AI of 300. Maximal distance between two neighboring lesions was ≤6 mm (a distance up to 7 mm was tolerated at the posterior wall to limit risk of esophageal injury). If PVI failed to restore SR, ECV was performed. If stable SR was achieved (≥60 s) after PVI and ECV, the procedure was terminated. If stable SR was not achieved following PVI and ECV, left atrial substrate modification (LASM) was performed by creating linear lesions, focal ablation, or both at the discretion of the operator. If linear lesions were created, conduction block of the created lines was required.

In the case of preprocedural or periprocedural documentation of a typical AFL, CTI ablation was performed until complete block was achieved [11,12]. Primary adverse events occurring within the first month after ablation were recorded.

### 2.5. Blanking Period

The duration of the blanking period (BP) was set at 3 months, and any recurrence of ATAs (>30 s) during this period was not considered a failure of the ablation procedure [13] and could be treated if required by introducing or increasing the dose of ADT, by performing a new ECV or a repeat ablation procedure.

If ECV was performed during the BP, the results and time between the ECV and the day of ablation were collected. If a repeat procedure was performed during the BP, the pulmonary vein reconnection (PVR) sites were ablated. At the operator’s discretion, LASM could be performed during the repeat procedure.

### 2.6. Follow-Up

Complications were recorded during a 12-month follow-up period. After ablation, anticoagulation and previously failed ADT were continued. At 3 months, anticoagulation was continued according to the CHA_2_DS_2_VAS_c_ score, whereas ADT was continued at the discretion of the treating physician. Clinical evaluation and electrocardiogram were performed at 1, 3, 6 and 12 months, or in cases of symptoms.

Recurrence was defined as any episode of AF, AT or AFL > 30 s on Holter electrocardiographs performed at 3, 6 and 12 months.

After a 3-month BP, 12-month freedom from AF and ATA recurrence was assessed. Freedom from AF and ATA recurrence were defined as freedom from any AF episode > 30 s and from any AF, AT or AFL episodes > 30 s, respectively.

In cases of documented ATA recurrence beyond the BP, the type of arrhythmia (AF, AT, or AFL) and the form of ATA recurrence (paroxysmal or persistent) were collected. Changes in ADT, ECV repetition and repeat ablation were also recorded.

### 2.7. Statistical Analysis

The normality of data distribution was tested using the Shapiro–Wilk test. Continuous variables were expressed as mean ± SD if normally distributed, medians with interquartile ranges if non-normally distributed, and dichotomous variables as percentages. The Kaplan–Meier curve was used to represent freedom from ATAs after 12 months of follow-up. Statistical significance was set at *p* < 0.05. All statistical analyses were performed using Microsoft^®^ Excel (Microsoft^®^, Washington, DC, USA).

## 3. Results

### 3.1. Population Selection

From the 55 consecutive patients referred for persistent AF ablation, five met the non-inclusion criteria (previous LA ablation, *n* = 1; ongoing uninterrupted AF episode ≥ 3 years, *n* = 1; mitral mechanical valve, *n* = 1; age > 80 years, *n* = 1; left ventricle ejection fraction ≤ 15%, *n* = 1). Finally, 50 patients were included in the study (La Timone University Hospital, *n* = 35; Pasteur University Hospital, *n* = 15)

### 3.2. Patient Characteristics

All patients had persistent AF (baseline clinical characteristics are shown in Table 1). Thirty-four patients underwent a scheduled ECV (68%) 56 ± 38 days prior to ablation. ECV was successful in 30 out of 34 patients (88%).

### 3.3. Procedural Characteristics

Patient characteristics are listed in Table 2. PVI was obtained for all patients in all circles. General anesthesia was used in 34 patients (68%). Mean procedure and fluoroscopy times were 141 ± 33 min and 7 ± 6 min, respectively. Total RF time was 23 ± 7 min. Rhythm at the beginning of the procedure was SR in 26 patients (52%), AF in 23 patients (46%) and CTI-dependent AFL in 1 patient (2%). After PVI, ECV was performed in 21 patients (42%). After PVI and ECV, LASM was required in one patient (2%); this procedure consisted of a posterior box creation followed by ECV (box isolation was confirmed after SR restoration). In six patients (12%), CTI ablation was performed with complete block. At the end of the procedure, stable SR was observed in all patients.

### 3.4. ECV during and after the BP

During the BP, eight patients (16%) experienced persistent AF recurrence, from which seven patients were receiving class I-III ADT; SR was restored in all eight patients by ECV performed 62 ± 17 days after the Ablation Index procedure. Out of the eight patients who underwent ECV during the BP, only three experienced persistent AF recurrence during the 9-months evaluation period. None of the patients underwent ECV after the BP.

### 3.5. Follow-Up Compliance

Of the 50 patients enrolled in the study, 50 (100%) completed the 12-month follow-up. Holter monitoring compliance was 90% at the 3-month visit, 90% at the 6-month visit, and 98% at the 12-month visit. In total, Holter monitoring compliance was 92.6% at the scheduled visits.

### 3.6. 12-Month Freedom from AF and AF/AT/AFL

**Figure 3:**
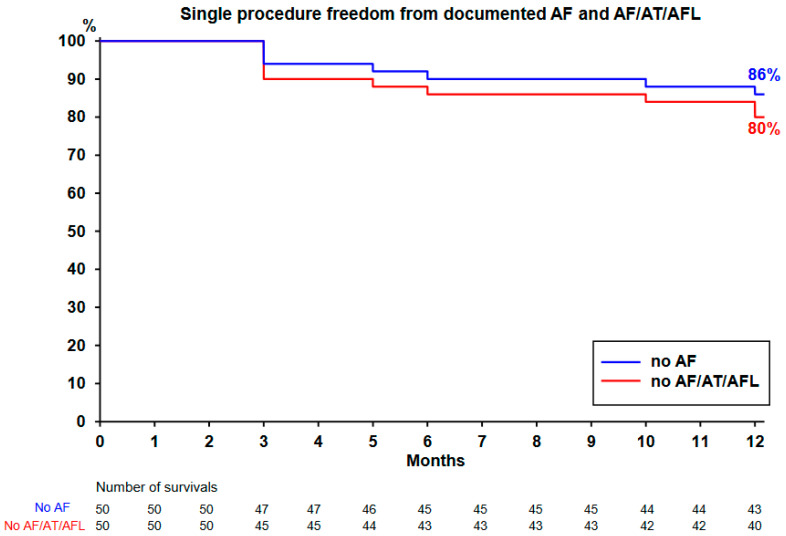
Survival plot after ablation. Kaplan-Meier curve depicting time to first recurrence of atrial fibrillation (AF), atrial tachycardia (AT), or atrial flutter (AFL), (including a 3-month blanking period) in all 50 patients undergoing CLOSE-guided ablation. At 12 months, 19 patients (38%) were still on anti-arrhythmic drug therapy.

**Figure 4:**
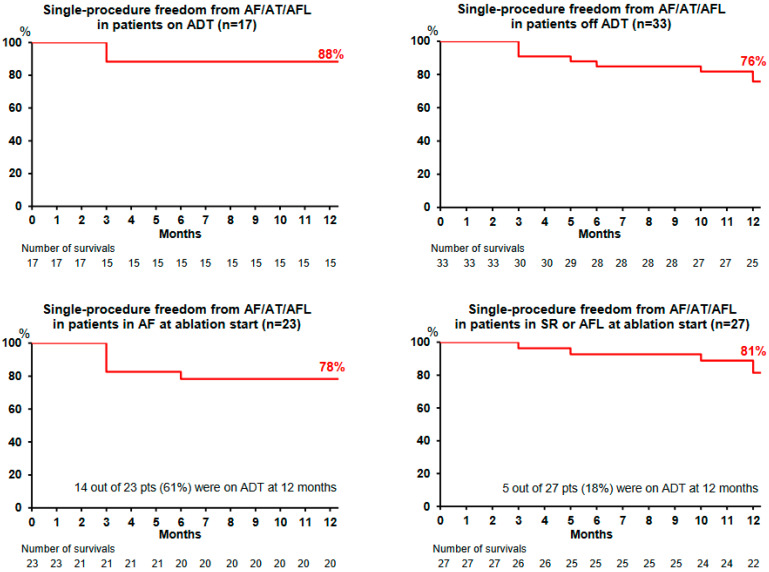
Survival plots for patient subgroups. Kaplan-Meier curves depicting time to first recurrence of AF (atrial fibrillation), AT (atrial tachycardia), or AFL (atrial flutter) in patients undergoing single procedure of CLOSE-guided PVI for subgroup of patients taking antiarrhythmic drug therapy (ADT) (**upper left**) or not taking ADT (**upper right**) and time to first recurrence of persistent AF, AT or AFL in patients in AF at ablation start (**lower left**) or in sinus rhythm (SR) of AFL at ablation start (**lower right**).

The Kaplan–Meier curve is plotted in Figure 3 and Figure 4. All the 50 patients completed the 12-month follow-up. Single-procedure 12-month freedom from AF and from AF/AT/AFL was, respectively, 86% and 80% (Figure 3). Throughout the course of the study, 19 patients (38%) remained on ADT (maintained in 17 patients and restarted in 2 other patients). Single-procedure freedom values from AF/AT/AFL in patients receiving ADT (*n* = 17) and off ADT (*n* = 33) were 88% (Figure 4, upper left) and 76% (Figure 4, upper right), respectively.

Single-procedure freedom from AF/AT/AFL recurrence in patients presenting with AF at the beginning of the ablation was 78% (Figure 4, lower left), with 14 out of 23 patients (61%) receiving ADT at 12 months. Single-procedure freedom from AF/AT/AFL recurrence in patients presenting with SR, AT, or AFL at the beginning of the ablation was 81% (Figure 4, lower right), with 5 out of 27 patients (18%) receiving ADT at the 12-month follow-up visit.

### 3.7. Arrhythmias Subtypes and ADT Evolution after Ablation

**Figure 5:**
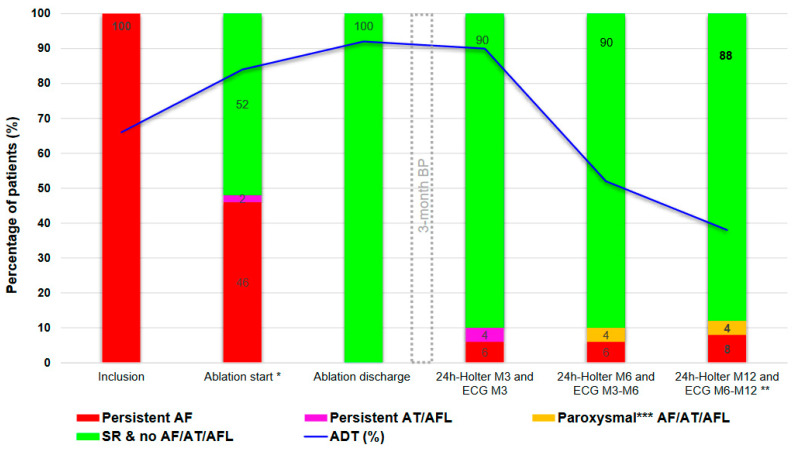
Subtypes of atrial tachyarrhythmia (>30 s) and ADT evolution throughout the study (*n* = 50). ADT = anti-arrhythmic drug therapy; AF = atrial fibrillation; AFL = atrial flutter; AT = atrial tachycardia; BP = blanking period; SR = sinus rhythm. * 34/50 pts underwent a scheduled electrical cardioversion (ECV) prior to ablation. ** ECV was performed only during the BP in 8 patients; no patient underwent repeat ablation. ADT was maintained in 38% of patients at 12 months. *** paroxysmal: AF, AT or AFL episode < 24 h on Holter ECG recording or 2 ECG performed within a 7 days period showing SR at least one time.

Arrhythmias subtypes and ADT after ablation are shown in Figure 5.

AF/AT/AFL recurrence was documented in 10 patients (20%) after the 3-month BP. Of the 10 patients with recurrence, 5 still received ADT at the time of recurrence. Paroxysmal ATAs were documented in four patients (8%), with mainly paroxysmal AF (in three patients). Persistent ATAs were documented in six patients (12%) at the 3-month follow-up: AF in four patients, AT in one patient, and typical AFL in one patient. In the two patients with persistent AT/AFL, spontaneous restoration of SR (without changes in ADT) was observed at the next scheduled visit, without any recurrence of ATA on Holter recordings performed at 6 and 12 months. In one patient who experienced persistent AF recurrence 10 months after ablation but did not receive ADT, SR was restored after flecainide reintroduction. For the three patients with persistent AF recurrence, repeat ablation was advised.

A total of 33 patients (66%) were on class I-III ADT at the time of inclusion; 46 patients (92%) were on class I-III ADT at ablation discharge. After the 3-month follow-up visit, the number of patients receiving ADT decreased to 19 (38%) at 12 months, including 2 patients (4%) for whom ADT was reinitiated or the ADT dose was increased due to ATA recurrence; of note, after ADT resumption or dose increase in the two patients mentioned, ATA recurrence was no longer documented during the rest of the follow-up period.

### 3.8. Ablation Repetition

Repeat ablation was indicated for three patients with persistent AF recurrence, but no repeat procedure was performed. One patient declined and, in the two other patients, repeat ablation was postponed for medical reasons (one patient was waiting for thyroidectomy before amiodarone resumption; the other patient had morbid obesity and maintained excessive alcohol intake requiring specific care before repeat ablation).

### 3.9. Primary Adverse Events

Two periprocedural complications were observed. One patient with a previous history of sinus node dysfunction presented with transient sinus node dysfunction after SR restoration, which resolved spontaneously within 24 h after ablation without pacemaker implantation (12 months after the Ablation Index procedure, pacemaker implantation was still not needed). One case of non-effusive pericarditis was observed on the day of ablation and was related to the procedure; the patient recovered completely after 4 weeks of oral treatment with colchicine.

No femoral vascular damage, stroke, transient ischemic attack, pericardial effusion, tamponade, atrioesophageal fistula, nor death were observed. Although neither pulmonary vein imaging nor chest radiography were systematically performed after ablation, none of the patients presented with suspicious symptoms indicative of pulmonary vein stenosis or phrenic nerve injury.

## 4. Discussion

### 4.1. Main Findings

Our study demonstrates that CLOSE-guided PVI performed in a population with persistent AF monitored using intermittent cardiac rhythm recordings is associated with a high 12-month single-procedure success rate (80%), with one third of the participants still receiving ADT at the end of the follow-up. The two-center nature of our study suggests that this approach may be reproducible using different electrophysiology staff and lab facilities.

In patients with recurrence of AF/AT/AFL after CLOSE-guided PVI, persistent AF and persistent AT/AFL were the dominant forms of recurrence; regardless of paroxysmal or persistent ATA, in 70% of patients with ATA recurrence, SR was restored either spontaneously or after resumption of ADT.

### 4.2. Candidates for CLOSE-Guided PVI

The studied population was representative of our practice (64-year-old, mostly male participants with obesity; median CHA2DS2VASc score 2.2 ± 1.7), mainly restricted to non-long standing persistent AF, with a relatively short duration of uninterrupted AF episode at the time of inclusion (4 months). Of note, 86% of patients had a history of previous ECV and our cohort presented markedly dilated LAs (50 mL/m^2^) related to electro-anatomical remodeling observed in advanced stages of persistent AF and associated with poor outcomes [14,15,16]. In a meta-analysis including populations with persistent AF who underwent various techniques of ablation, single-procedure success without ADT was 43% and increased up to 69% after multiple ablations and/or ADT dose increase, while the addition of extra-pulmonary substrate approaches was associated with declining efficacy when compared to PVI alone [17]. The high 12-month single-procedure success rate (80%) observed in our study may be explained by factors such as the non-inclusion of patients with severe structural heart disease (mitral valve surgery or severe left ventricle dysfunction) or the use of an optimized PVI approach (CLOSE-guided PVI), including ADT maintenance in one third of our cohort at the end of the follow-up.

### 4.3. One-Year Freedom from ATA

A summary of previous studies evaluating the effectiveness of CLOSE-guided PVI is shown in Table 3.

In a population with persistent AF and clinical characteristics comparable to our population who was treated with a similar protocol, Hussein et al. reported 80% 1-year freedom from 30 s AF/AT episodes using Holter recordings and daily electrocardiogram monitoring [18], in line with our results. However, in contrast to the study by Hussein et al., where patients with long-standing persistent AF were not included and a second ablation performed in 22% of patients, our study evaluated the effectiveness of a single CLOSE-guided PVI procedure in a population with persistent AF, including long-standing persistent AF. In a prospective study evaluating midterm efficacy of AI-guided PVI also using a similar protocol, Solimene and colleagues reported 78% freedom from AF/AT in a subgroup of 24 patients with persistent AF monitored with 24 h Holter recordings [19], also in line with our results. Nonetheless, patients with long-standing persistent AF were not included in that study, and not all the patients completed a 12-month follow-up period, which may have improved the outcomes. In a retrospective monocentric single-arm study evaluating the effectiveness of an AI-guided PVI strategy in a population with persistent AF including long-standing persistent AF, Yamaguchi et al. reported 70% freedom from AF/AT in patients not receiving ADT [20]; however, one quarter of the participants was followed up for only 7 months, which may have contributed to improved ablation outcomes.

In a multicenter registry evaluating the effectiveness of CLOSE-guided PVI in a population with persistent AF, Stabile et al. reported 83% freedom from AF/AT in patients receiving or not receiving ADT. However, in contrast to the study by Solimene et al., where patients with long-standing persistent AF were not included and rhythm at the start was AF in less than 13% of the studied population [21], patients with long-standing persistent AF were included in our study and almost half of the studied population was in AF at the beginning of the procedure, which are factors associated with a higher risk for atrial tachyarrhythmia recurrence.

Finally, our study is the first prospective, bicentric, single-arm study to evaluate the effectiveness of a single CLOSE-guided PVI procedure in a population with persistent AF, including long-standing persistent AF, during a follow-up period of at least 12 months for all patients.

### 4.4. Safety of CLOSE-Guided PVI

In our study, the rate of adverse events related to the procedure was low, reaching 2% (one case of pericarditis related to the ablation). The procedure time and radiation exposure remained acceptable for daily practice. Recently, a more extensive ablation strategy based on bi-atrial anatomical lesion sets including PVI + ligament of Marshall ablation + mitral line + roof line + CTI ablation was evaluated in a prospective monocentric single-arm study. The authors reported single-procedure 12-month freedom from AF/AT without ADT reaching 72% but, in contrast with our study, complications were observed in 8% of patients (two transient ischemic attack and four pericarditis cases), with prolonged procedure time (more than 4 h) and longer fluoroscopy time [22].

### 4.5. ADT throughout the Study

In our study, the use of ADT mildly decreased between time of inclusion (66%) and end of the follow-up period (38% at 12 months). In patients with paroxysmal AF, maintenance of ADT up to 9 and 12 months after RF-based standardized PVI («hybrid» rhythm control strategy) has been shown to improve clinical outcomes in a randomized controlled trial [23]. In our study of a population with persistent AF, 1-year single-procedure arrhythmia survival was slightly higher in patients receiving ADT. However, the impact of ADT maintenance after ablation on clinical outcomes in patients with persistent AF is still unknown and is under evaluation in a multicenter prospective randomized trial [24]. Concerns exist about safety in the case of long-term use of class I-III ADT [25], especially amiodarone [26], which should be stopped as soon as possible after individualized medical evaluation.

### 4.6. PVI as Ablation Index Strategy in Persistent AF Population

In the multicenter CRYO4PERSISTENT AF study that evaluated the efficacy of single-cryoballoon PVI-only ablation in a population with persistent AF comparable to our population (mainly non-long-standing persistent AF with mild LA dilation) monitored with Holter recordings, the authors reported ≈60% single-procedure freedom from ATA [27]. However, in our study, a higher proportion of patients received ADT than that in the CRYO4PERSISTENT AF study, which may explain the differences in the observed outcomes. In a multicenter randomized study comparing laser balloon-guided PVI with non-standardized RF-guided PVI in patients with non-long-standing persistent AF with clinical characteristics comparable to our study population, the authors reported 71% single-procedure freedom from ATA using Holter recordings [28], slightly inferior to the outcomes observed in our study, which may be related to longer AF episode duration in the population treated with laser balloon-guided PVI.

### 4.7. Limitation of Radiation Exposure

Owing to the use of an electro-anatomical mapping system, the fluoroscopy time was relatively low in our study (7 min), at least half of that reported in previously published studies that evaluated the efficacy of cryoballoon-based PVI in a persistent AF population [29,30]. Andreassi et al. reported that the odds ratios for developing cancer and cataracts were 3 and 6.3, respectively, in a study including 218 electrophysiologists compared to non-exposed professionals [31]. More recently, it has been reported that the lifetime attributable risk for all cancer incidence is 0.4% for male and 1.5% for female electrophysiologists, while the risk for cancer mortality is 0.22% for male and 0.83% for female electrophysiologists [32]. When considering the lifetime exposure of an electrophysiologist, this may be a critical concern for reducing the risk of X-ray irradiation-related diseases. Thus, among the available AF ablation technologies presenting similar safety and efficacy, those associated with lower radiation exposure should be preferred for the benefit of the patients as well as for the exposed professionals.

### 4.8. Impact on Outcomes of Rhythm at the Beginning of the Ablation Procedure

Single-procedure freedom from AF/AT/AFL recurrence was similar between patients with AF at the beginning of the ablation procedure and those in SR, AT or AFL (78% vs. 81%), in contrast to a previous persistent AF ablation study where the authors reported that SR at the beginning of the procedure was associated with better outcomes compared to AF [33]. In our study, patients presenting with AF at the beginning of the ablation procedure had a 3-fold higher rate of ADT use at the end of the follow-up compared to those in SR or with AT or AFL, which may have contributed to the lack of difference in observed outcomes. Whether rhythm at the beginning of the ablation procedure is a prognostic marker of success needs to be assessed in further studies on ADT withdrawal at the end of the BP.

Finally, our strategy consisting of a single CLOSE-guided PVI procedure in a population with mainly non-long-standing persistent AF was efficient in maintaining stable SR.

## 5. Study Limitations

This study had a single-arm design and included only a limited number of patients. However, this study prospectively evaluated the largest population of patients with persistent AF, including long-standing persistent AF, with a complete 12-month follow-up period, treated using an ablation strategy limited to CLOSE-guided PVI.

In our study, arrhythmia recurrence was assessed using 24 h Holter recordings, which led to undiagnosed ATA recurrence, and thus overestimation of success [34].

Although representative of the patients referred to our centers for ablation, the patients included in this study mainly presented non-long-standing persistent AF, which precludes generalization for patients with long-standing persistent AF.

These limitations could be addressed by a large-scale randomized controlled trial, with 30 day Holter monitoring or an insertable cardiac monitor.

## 6. Conclusions

In a population with persistent AF monitored with intermittent cardiac rhythm recordings, CLOSE-guided PVI was associated with a high 1-year single-procedure arrhythmia survival rate. In this population, one out of six patients required ECV during the BP to terminate persistent AF recurrence and ADT was maintained in one third of the cases. Large-scale multicenter studies focusing on ATA burden monitored using insertable cardiac monitors are required.

## 7. Contribution to the Field

CLOSE-guided PVI may be a first-line ablation strategy for treating persistent AF, particularly in patients with non-long-standing persistent AF. CLOSE-guided PVI is a non-extensive, safe, and reproducible ablation approach that provides good clinical outcomes 1 year after ablation in a population with persistent AF.

## Figures and Tables

**Table 1:** Patient Characteristics (*n* = 50).

Age, yrs	64 ± 10
Male	40 (80)
BMI, kg/m^2^	30.5 ± 4.8
Arterial hypertension	25 (50)
Diabetes	10 (20)
CAD	7 (14)
CHA2DS2VASc score, pts	2.2 ± 1.7
Indexed left atrial volume, mL/m^2^	50 ± 19
LVEF, %	55 ± 9
Persistent AF	50 (100)
Long-standing * persistent AF	7 (14)
AF history, months	11 (4, 31)
Continuous AF episode duration, months	4 (3, 9)
History of ECV	43 (86)
ECV before inclusion, number	1.3 ± 0.8
Scheduled ECV within 6 months prior to ablation, pts	34 (68)
Time between scheduled ECV to ablation, days	56 ± 38
Failure of ≥1 class I-III ADT	34 (68)
Intolerance or unwillingness to take long-term ADT	16 (32)
Class I-III ADT on the day of ablation	42 (84)
Amiodarone	40 (80)
Other class I-III ADT	2 (4)

Values are *n* (%), mean ± SD, or median (interquartile range). * ongoing continuous AF episode > 1 year, ADT = antiarrhythmic drug therapy; AF = atrial fibrillation; BMI = body mass index; CAD = coronary arterial disease; CHA2DS2VASc: Congestive heart failure, Hypertension, Age ≥ 75 years, Diabetes mellitus, Stroke/transient ischemic attack/thromboembolism, Vascular disease, Age 65 to 74 years, Sex category; ECV = electrical cardioversion; LVEF = left ventricle ejection fraction.

**Table 2:** Procedure Characteristics (*n* = 50).

PV isolation	50 (100)
Left PVs first pass isolation	46 (92)
Right PVs first pass isolation	39 (78)
CTI ablation	6 (12)
General anaesthesia	34 (68)
Esophageal thermal probe	10 (20)
Procedure time, min	141 ± 33
Radiofrequency time, min	23 ± 7
Fluoroscopy time, min	7 ± 6
Rhythm at start of procedure	
SR	26 (52)
AF	23 (46)
CTI-dependent AFL	1 (2)
SR restoration	
During PVI or CTI ablation	2 (4)
After PVI and ECV	21 (42)
After PVI, LA posterior box and ECV	1 (2)
Stable SR at end of procedure	50 (100)

Values are *n* (%), mean ± SD, or median (interquartile range). AF = atrial fibrillation; AFL = atrial flutter; CTI = cavotricuspid isthmus; ECV = electrical cardioversion; LA = left atrium; PV = pulmonary veins; RF = radiofrequency; SR = sinus rhythm.

**Table 3:** Summary of previous studies evaluating effectiveness of CLOSE-guided PVI in persistent atrial fibrillation.

	PRAISE 2018, *n* = 40	Solimene 2019, *n* = 32	Yamaguchi 2020, *n* = 140	AIR Registry 2020, *n* = 96	Our Study, *n* = 50
Design	Prospective, bicentric, single-arm	Prospective, monocentric, single-arm	Prospective, multicenter, single-arm	Retrospective, monocentric, single-arm	Prospective, multicenter, single-arm
LS-persistent AF (%)	0	0	35 (25)	0	7 (14)
BMI, kg/m^2^	n/a	n/a	25.3 ± 4.1	n/a	30.5 ± 4.8
CHA2DS2VASc, pts	1 (0–2)	n/a	2 (1–2)	n/a	2 (1–3)
Arterial hypertension (%)	12 (30)	n/a	77 (55)	n/a	25 (50)
AF at the beginning of the ablation (%)	n/a	n/a	n/a	n/a (≤13.3)	23 (46)
AF episode duration, months	9.5 (6–12)	n/a	6 (3.6–13.2)	n/a	4 (3–9)
Left atrial diameter, mm	43 ± 5	n/a	43 ± 6	n/a	n/a
Left atrial volume, mL/m^2^	n/a	n/a	n/a	n/a	50 ± 19
Ablation strategy	CLOSE-guided PVI (AI 550 AW, 400 PW; ITD 6 mm); PV re-isolation at 3 months in 22% of patients	Single procedure of CLOSE-guided PVI (AI 400–450 AW, 330–350 PW; ITD 6 mm)	Ablation Index-guided PVI (AI 500 AW, 400–500 PW; ITD 6 mm)	Single procedure of CLOSE-guided PVI (AI 500 AW, 300 PW or AI 450 AW, 330 PW; ITD 6 mm)	Single procedure of CLOSE-guided PVI (AI 450 AW, 300 PW; ITD 6 mm)
Arrhythmia monitoring	12-lead ECG, 24-h Holter at 3, 6 and 12 months; TTM	12-lead ECG, 24-h Holter at 3, 6 and 12 months	12-lead ECG, 24-h Holter at 3, 6 and 12 months	12-lead ECG, 24-h Holter at 3, 6 and 12 months	12-lead ECG, 24-h Holter at 3, 6 and 12 months
≥12-month FU for all patients	Yes	No	No	No	Yes
12-month AF/AT freedom on ADT (%)	n/a	n/a	n/a	n/a	88
12-month AF/AT freedom off or on ADT (%)	80	78	n/a	83	80
12-month AF/AT freedom off ADT (%)	n/a	67	70	n/a	76
Proportion of population on ADT at 12-month (%)	10	n/a	0	26	38

ADT = antiarrhythmic drug therapy; AF = atrial fibrillation; AI= Ablation Index; AT = atrial tachycardia; AW = anterior wall; ECG = electrocardiogram; FU = follow-up; ITD = intertag distance; LS = long standing; PVI = pulmonary veins isolation; PVs = pulmonary veins; PW = posterior wall; TTM = transtelephonic monitor.

## Data Availability

The data presented in this study are available on request from the corresponding author. The data are not publicly available due to privacy.

## Abbreviations

AAD	anti-arrhythmic drug
ADT	anti-arrhythmic drug therapy
AF	atrial fibrillation
AFL	atrial flutter
AI	Ablation Index
ASM	atrial substrate modification
AT	atrial tachycardia
ATA	atrial tachyarrhythmia
CCW	counterclockwise
CF	contact force
CFAE	complex fractionated atrial electrograms
CIED	cardiac implantable electronic device
CTI	cavotricuspid isthmus
ECG	electrocardiogram
ECV	electrical cardioversion
EGM	electrogram
ICM	insertable cardiac monitor
LA	left atrium/atrial
LOM	ligament of Marshall
LVEF	left ventricle ejection fraction
PV	pulmonary vein
PVI	pulmonary vein isolation
PVR	pulmonary vein reconnection
RF	radiofrequency
SR	sinus rhythm
TIA	transient ischemic attack
